# On the Analysis of Genome-Wide Association Studies in Family-Based Designs: A Universal, Robust Analysis Approach and an Application to Four Genome-Wide Association Studies

**DOI:** 10.1371/journal.pgen.1000741

**Published:** 2009-11-26

**Authors:** Sungho Won, Jemma B. Wilk, Rasika A. Mathias, Christopher J. O'Donnell, Edwin K. Silverman, Kathleen Barnes, George T. O'Connor, Scott T. Weiss, Christoph Lange

**Affiliations:** 1 Department of Statistics, Chung-Ang University, Seoul, Korea; 2 Research Center for Data Science, Chung-Ang University, Seoul, Korea; 3 Department of Neurology, Boston University School of Medicine, Boston, Massachusetts, United States of America; 4 Genometrics Section, Inherited Disease Research Branch, National Human Genome Research Institute, National Institutes of Health, Baltimore, Maryland, United States of America; 5 National Heart, Lung, and Blood Institute and Framingham Heart Study, Bethesda, Maryland, United States of America; 6 Cardiology Division, Massachusetts General Hospital, Harvard Medical School, Boston, Massachusetts, United States of America; 7 Channing Laboratory, Department of Medicine, Brigham and Women's Hospital, Boston, Massachusetts, United States of America; 8 Division of Pulmonary and Critical Care Medicine, Brigham and Women's Hospital, Boston, Massachusetts, United States of America; 9 Harvard Medical School, Boston, Massachusetts, United States of America; 10 Department of Medicine, School of Medicine, Johns Hopkins University, Baltimore, Maryland, United States of America; 11 Pulmonary Center, Boston University School of Medicine, Boston, Massachusetts, United States of America; 12 Center for Genomic Medicine, Brigham and Women's Hospital, Boston, Massachusetts, United States of America; 13 Department of Biostatistics, Harvard School of Public Health, Boston, Massachusetts, United States of America; University of California San Diego and The Scripps Research Institute, United States of America

## Abstract

For genome-wide association studies in family-based designs, we propose a new, universally applicable approach. The new test statistic exploits all available information about the association, while, by virtue of its design, it maintains the same robustness against population admixture as traditional family-based approaches that are based exclusively on the within-family information. The approach is suitable for the analysis of almost any trait type, e.g. binary, continuous, time-to-onset, multivariate, etc., and combinations of those. We use simulation studies to verify all theoretically derived properties of the approach, estimate its power, and compare it with other standard approaches. We illustrate the practical implications of the new analysis method by an application to a lung-function phenotype, forced expiratory volume in one second (FEV1) in 4 genome-wide association studies.

## Author Summary

In genome-wide association studies, the multiple testing problem and confounding due to population stratification have been intractable issues. Family-based designs have considered only the transmission of genotypes from founder to nonfounder to prevent sensitivity to the population stratification, which leads to the loss of information. Here we propose a novel analysis approach that combines mutually independent FBAT and screening statistics in a robust way. The proposed method is more powerful than any other, while it preserves the complete robustness of family-based association tests, which only achieves much smaller power level. Furthermore, the proposed method is virtually as powerful as population-based approaches/designs, even in the absence of population stratification. By nature of the proposed method, it is always robust as long as FBAT is valid, and the proposed method achieves the optimal efficiency if our linear model for screening test reasonably explains the observed data in terms of covariance structure and population admixture. We illustrate the practical relevance of the approach by an application in 4 genome-wide association studies.

## Introduction

During the analysis phase of genome-wide association studies, one is confronted with numerous statistical challenges. One of them is the decision about the “right” balance between maximization of the statistical power and, at the same time, robustness against confounding. In family-based designs, the possible range of analysis options spans from a traditional family-based association analysis [1]–[4], e.g. TDT, PDT, FBAT, to the application of population-based analysis methods that have been adapted to family-data [1]–[3]. While, by definition, the first group of approaches is completely immune to population admixture and model misspecification of the phenotype, and can be applied to any phenotype that is permissible in the family-based association testing framework (FBAT [4]–[6]), the second category of approaches maximizes the statistical power by a population-based analysis. The phenotypes are modeled as a function of the genotype, and population-based methods such as genomic control [7],[8], STRUCTURE [9] and EIGENSTRAT [10], are applied to account for the effects of population admixture and stratification. Hybrid-approaches that combine elements of both population-based and family-based analysis methods, e.g. VanSteen algorithm [11] and Ionita weighting-schemes [12],[13] have been suggested to bridge between the 2 types of analysis strategies. Contrary to the other methods that combine family data and unrelated samples [14]–[17], such hybrid testing strategies maintain the 2 key features of the family-based association tests: The robustness against confounding due to population admixture and heterogeneity, and the analysis flexibility of the approach with respect to the choice of the target phenotype. Such 2-stage testing strategies utilize the information about the association at a population-level, the between-family component, to prioritize SNPs for the second step of the approach in which they are tested formally for association with a family-based test. The hybrid approaches can achieve power levels that are similar to approaches in which standard population-based methods are applied to family-data, but the optimal combination of the 2 sources of information (the between-family component and the within-family component) is not straightforward in the hybrid approaches.

In this communication, we propose a new family-based association test for genome-wide association studies that combines all sources of information about association, the between and the within-family information, into one single test statistic. The new test is robust against population-admixture even though both components, the between and the within-family components, are used to assess the evidence for association. The approach is applicable to all phenotypes or combinations of phenotypes that can be handled in the FBAT-approach, e.g. binary, continuous, time-to-onset, multivariate, etc [4]–[6],[18]. While the correct model specification for the phenotypes will increase the power of the proposed test statistic, misspecification of the phenotypic model does not affect the validity of the approach. Using extensive simulation studies, we verify the theoretically derived properties of the test statistic, assess its power and compare it with other standard approaches. An application to the Framing heart study (FHS) illustrates the value of the approach in practice. A new genetic locus for the lung-function phenotype, FEV1 (forced expiratory volume in the first second) is discovered and replicated in 3 independent, genome-wide association studies.

## Methods

We assume that in a family-based association study, *n* family members have been genotyped at *m* loci with a genome-wide SNP-chip. For each marker locus, a family-based association test is constructed based on the offspring phenotype and the within-family information. The within-family information is defined as the difference between the observed, genetic marker score and the expected, genetic marker score, which is computed conditional upon both the parental genotypes/sufficient statistic [19] under the assumption of Mendelian transmissions. We denote the family-based association test for the *i*th marker locus by *FBAT_i_*. Such an FBAT statistic can be the standard TDT, an FBAT for quantitative/qualitative traits, FBAT-GEE for multivariate traits, etc [4],[6],[18],[20],[21]. Similarly, for the *i*th marker, the between-family information can be used to assess the evidence for association at a population-based level by computing a VanSteen-type [11] “screening statistic” *T_i_*. The screening statistic is computed based on the data for offspring phenotype and the parental genotypes/sufficient statistic. The statistic *T_i_* can be a Wald test for the genetic effect size that is estimated based on the conditional mean model [22], or the estimated power of the family-based test *FBAT_i_* [23], either of which is feasible. However, while the *FBAT_i_* statistic is robust against population stratification, the screening statistic *T_i_* is susceptible to confounding. For this reason, the VanSteen-type testing strategies have restrictively used the between-family information as weights for p-values of the *FBAT-*statistic, but never as a component in the test statistic itself.

### Construction of an overall family-based association test including the population-based and family-based components

In order to construct a family-based association test that incorporates both the within and the between-family information, the Z-statistics that correspond to the p-values of *FBAT_i_* and *T_i_* are computed. The statistic Z_α_^*^ is the *α* quantile of standard normal distribution. *pFBAT_i_* and *pT_i_* are the p-value of the FBAT-statistics and one sided p-value of the screening statistic where the direction of the one sided screening statistic is defined by the directionality of *FBAT_i_*. Based on the statistical independence of *FBAT_i_* and *T_i_* [11] under the null-hypothesis, we can obtain an overall family-based association test statistic *Z_i_* by combining both Z-statistics in a weighted sum, 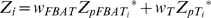
where the parameters *w_FBAT_* and *w_T_* are standardized weights so that the overall family-based association test *Z_i_* has a normal distribution with mean 0 and variance 1, i.e. *w_FBAT_* ^2^+*w_T_* ^2^ = 1. In the literature, this approach of combining two test statistics is known as the Liptak-method [24]. However, the Liptak-method varies here from its standard application in that the 2 test statistics have to be combined so that confounding in the screening statistic *T_i_* cannot affect the validity of the overall family-based association test statistic *Z_i_*. In the context of a genome-wide association study (GWAS), we are able to achieve this goal by using rank-based p-values for the screening statistic *T_i_* instead of their asymptotic p-values.

The “screening statistics” *T_i_* are sorted based on their evidence for association so that *T*_(*m*)_ denotes the screening statistic with the least amount of evidence for association and *T*_(1)_ the screening statistic with the largest amount of evidence for association. The rank-based p-value, (*i* – 0.5)/*m*, is then assigned to the ordered screening test statistics *T*_(*i*)_. If there is a tie, then the average of the ranks will be used for the computation of the rank-based p-value for the *i*th marker. Since the null-hypothesis will be true for the vast majority of the SNPs in a GWAS, the rank-based p-values provide an alternative way to assess the significance of the population-based screening statistic *T_i_*. The overall association test *Z_i_* is then computed based on the Z-score for the asymptotic p-value of the FBAT-statistic and the *Z*-score for the ranked-based p-value of the screening statistic *T_i_*. In Text S1 we show that the overall association test *Z_i_* maintains the global significance level *α*, under any situations including population admixture and stratification. This can be understood intuitively as well. The smallest rank-based p-value is 0.5/*m*. Using the Bonferroni-correction to adjust for multiple testing, the individual, adjusted significance level is *α*/*m* which will always be smaller than the smallest rank-based p-value, 0.5/*m*, unless the pre-specified global significance level *α* is great than 0.5. This implies that the overall family-based association test can never achieve genome-wide significance just based on the rank-based p-values alone. The FBAT-statistic has to contribute evidence for the association as well in order for the overall family-based association test to reach genome-wide significance. Finally, we have to address the specification of the weights *w_FBAT_* and *w_T_* in the overall family-based association test statistic *Z_i_*. While any combination of weights *w_FBAT_* and *w_T_* will provide a valid test statistic *Z_i_*, the most powerful overall statistic *Z_i_* is approximately achieved when the ratio of the weights is equal to the ratio of the standardized effect sizes, the expected effect size of the regression coefficient divided by its (estimated) standard deviation. For quantitative traits in unascertained samples, one can show that optimal power levels are achieved for equal weights, i.e. *w_FBAT_* = *w_T_*. In general, the equal weighting scheme seems to provide good power levels for any disease mode of inheritance and for different trait types, e.g. binary traits, time-to-onset, etc. The theoretical derivation of optimal weighting schemes for such scenarios is ongoing research and will be published subsequently.

Furthermore, it is important to note that, instead of the Liptak-method, Fisher's method for combining p-values could have been used as well to construct an overall family-based association test which would have the same robustness properties as the overall-test based on the Liptak-method. However, simulation studies (data not shown) suggest that the highest power levels are consistently achieved with the Liptak method. We therefore omit the approach based on Fisher's method here.

## Results

### Type I error for 500K GWAS

In the first part of the simulation study, the type-1 error of the proposed family-based association test denoted as LIP was assessed in the absence and in the presence of population admixture, and we use the Wald test based on the conditional mean model [22] with between-family component for *pT_i_* in our all simulations. For various scenarios, we verified that the proposed overall family-based association test maintains the *α*-level.

For simplicity, we assume in the simulation studies that the random samples are given, i.e. no ascertainment, and that the parental genotypes are known. Assuming Hardy-Weinberg equilibrium, the parental genotypes are generated by drawing from Bernoulli distributions defined by the allele frequencies. The offspring genotypes are obtained by simulated Mendelian transmissions from the parents to the offspring. For the *j*th trio, the offspring phenotype *Y_j_* is simulated from a Normal distribution with mean *aX_j_* and variance 1, i.e. *N*(*aX_j_*, 1), where the parameter *a* represents the genetic effect size and the variable *X_j_* denotes the offspring genotype. Under the null-hypothesis of no association, the genetic effect size parameter *a* will be set to 0.

For scenarios in which population admixture is present, we assume that the admixture is created by the presence of 2 subpopulations whose phenotypic means differ by 0.2. The allele frequencies for each marker in the two subpopulations are generated by the Balding-Nichols model [25]. That is, for each marker, the allele frequency in an ancestral population is generated from a uniform distribution between 0.1 and 0.9, *U*(0.1, 0.9). Then, the marker allele frequencies for the two subpopulations are independently sampled from the beta distributions (*p*(1−*F_ST_*)/*F_ST_*, (1−*p*)(1−*F_ST_*)/*F_ST_*) for the whole markers in each replicate of the simulated GWAS. A survey reported *F_ST_* estimates with a median of 0.008 and 90*th* percentile of 0.028 among Europeans, and the corresponding values are 0.027 and 0.14 among Africans, and 0.043 and 0.12 among Asians [26]. The value for Wright's *F_ST_* was assumed to be 0.05, 0.1, 0.2, or 0.3. Each trio was assigned to the one of the 2 subpopulations with 50% probability.

In the absence and presence of the population stratification (*F_ST_* = 0.05, 0.1, 0.2, and 0.3), Table 1 shows the empirical type-1 error rates of the overall association test statistic *Z_i_* for a GWAS with 500,000 SNPs. The estimates for the empirical significance levels in Table 1 are based on 2,000 replicates. The empirical genome-wide significance level is calculated as the proportion of replicates for which the minimum p-values among the 500,000 markers is less than 0.05/500,000. We consider the proposed equal weights for *w_FBAT_* and *w_T_*, for genome-wide significance level 0.05 and Table 1 shows that the type-1 error rate is preserved well. For different significance levels, we calculate in Table 2 the empirical proportions of SNPs for which the overall family-based association test *Z_i_* is significant at the *α*-levels of 0.05, 0.01, 10^−3^, 10^−4^ and 10^−5^. The simulation studies are conducted in the absence and in the presence of population admixture. Table 2 does not provide any evidence for a departure of the empirical significance levels from the theoretical levels, both in the absence and presence of population substructure. These results confirm our theoretical conclusions that *Z_i_* is robust against population stratification and maintains correct type-1 error.

**Table 1:** Empirical type-1 error for 500K GWAS at genome-wide significance level 0.05.

*F_ST_*	Empirical error rate
0.00	0.0505
0.05	0.0395
0.10	0.0425
0.20	0.0450
0.30	0.0445

The number of trios, *N_trio_*, is assumed to be 1,000 and the empirical type-1 error of the minimum p-value for GWAS at 500K GWAS is calculated with 2,000 replicates.

**Table 2:** Average of empirical proportion at 500K GWAS.

*F_ST_*	*c* = 5×10^−2^	*c* = 1×10^−2^	*c* = 1×10^−3^	*c* = 1×10^−4^	*c* = 1×10^−5^
0.00	5.00×10^−2^	9.97×10^−3^	9.91×10^−4^	9.86×10^−5^	9.66×10^−6^
0.05	5.00×10^−2^	9.97×10^−3^	9.91×10^−4^	9.85×10^−5^	9.76×10^−6^
0.10	5.00×10^−2^	9.96×10^−3^	9.88×10^−4^	9.78×10^−5^	9.79×10^−6^
0.20	4.99×10^−2^	9.95×10^−3^	9.87×10^−4^	9.76×10^−5^	9.60×10^−6^
0.30	4.98×10^−2^	9.92×10^−3^	9.82×10^−4^	9.68×10^−5^	9.40×10^−6^

The number of trios, *N_trio_*, is assumed to be 1,000 and the empirical proportions of SNPs whose p-values for *Z_i_* are less than *c* in each replicate for 500K GWAS are averaged over 2,000 replicates.

In the next set of simulation studies, we assess the effects of the local population stratification on the overall family-based association test. We generate local population stratification under the following assumptions: there are two subpopulations, *G*_1_ and *G*_2_ which distinguish themselves from each other in 2 marker regions. We assume that a subject can be from all possible 4 combinations at the 2 particular regions, e.g. (*G*_1_, *G*_1_), (*G*_1_, *G*_2_), (*G*_2_, *G*_1_) and (*G*_2_, *G*_2_). Both regions consist of 10K SNPs and 90K SNPs respectively and if subjects are from the same subpopulation in each genetic region, their assumed allele frequencies of the markers in the corresponding region are equal. For example, the allele frequencies of each marker in the marker region 1 are the same for samples in (*G*_1_, *G*_1_) and (*G*_1_, *G*_2_), but they are different for (*G*_1_, *G*_1_) and (*G*_2_, *G*_2_). In the simulation study, we generate the parental genotypes based on these allele frequency assumptions and obtain the offspring genotypes based on simulated Mendelian transmissions. Using the Balding-Nichols model we considered *F_ST_*'s of 0.001, 0.005, 0.01 and 0.05 in the simulation studies. The offspring's phenotype was generated under the null hypothesis, but we assumed that each sub-population strata had a different phenotypic mean: 0 for (*G*_1_, *G*_1_), 0.2 for (*G*_1_, *G*_2_), 0.4 for (*G*_2_, *G*_1_) and 0.6 for (*G*_2_, *G*_2_). Each replicate consists of 2,000 trios with equal number of trios for all 4 possible combinations. The data was analyzed with the proposed overall family-based association test and with standard linear regression after adjusting population admixture with EIGENSTRAT [10]. For EIGENSTRAT, we applied the principal component analysis to the mean of the paternal and maternal genotypes at each locus because parents of each offspring are from the same subpopulation, and then the residuals obtained from regressing offspring genotypes and phenotypes with eigenvectors respectively are used to calculate the generalized Armitage trend test [27]. Table 3 provides the empirical type-1 error for both analysis approaches based on 2,000 replicates. While EIGENSTRAT exhibits an inflated type-1 error, the proposed overall family test maintains the theoretical significance level.

**Table 3:** Average of empirical proportion at 100K GWAS.

Method	*F_ST_*	*c* = 5×10^−2^	*c* = 1×10^−2^	*c* = 1×10^−3^	*c* = 1×10^−4^	*c* = 1×10^−5^
EIGENSTRAT	0.001	5.07×10^−2^	1.02×10^−2^	1.04×10^−3^	1.05×10^−4^	1.02×10^−5^
	0.005	5.44×10^−2^	1.17×10^−2^	1.36×10^−3^	1.72×10^−4^	2.45×10^−5^
	0.01	5.86×10^−2^	1.39×10^−2^	2.09×10^−3^	3.62×10^−4^	7.57×10^−5^
	0.05	8.20×10^−2^	3.24×10^−2^	1.32×10^−2^	6.58×10^−3^	3.39×10^−3^
LIP	0.001	5.00×10^−2^	9.99×10^−3^	9.93×10^−4^	9.89×10^−5^	9.70×10^−6^
	0.005	5.00×10^−2^	9.99×10^−3^	1.00×10^−3^	1.01×10^−4^	1.00×10^−5^
	0.01	5.00×10^−2^	9.99×10^−3^	9.97×10^−4^	9.96×10^−5^	9.99×10^−6^
	0.05	5.00×10^−2^	9.98×10^−3^	9.94×10^−4^	9.89×10^−5^	9.98×10^−6^

The number of trios, *N_trio_*, is assumed to be 1,000. The empirical proportions of SNPs whose p-values for *Z_i_* are less than *c* in each replicate for 500K GWAS are averaged over 2000 replicates when there is local population stratification. LIP stands for the proposed method using Liptak method to combine *pFBAT_i_* and *pT_i_*.

### Empirical power with simulation for 500K GWA for quantitative trait

**Figure 1:**
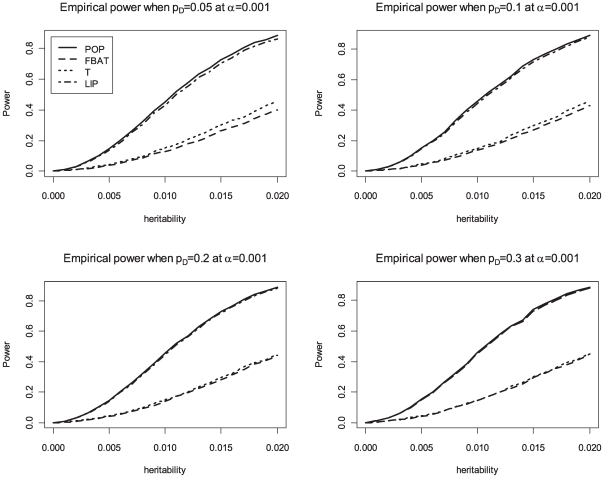
Empirical power at 0.001 significance level for additive disease. POP is the empirical power of the standard population-based method. T is the empirical power of the Wald test based on the conditional mean model [22] for between-faimly components. LIP is the empirical power of the combined p-values with Liptak's method. In this figure, FBAT and T are completely overlapped.

**Figure 2:**
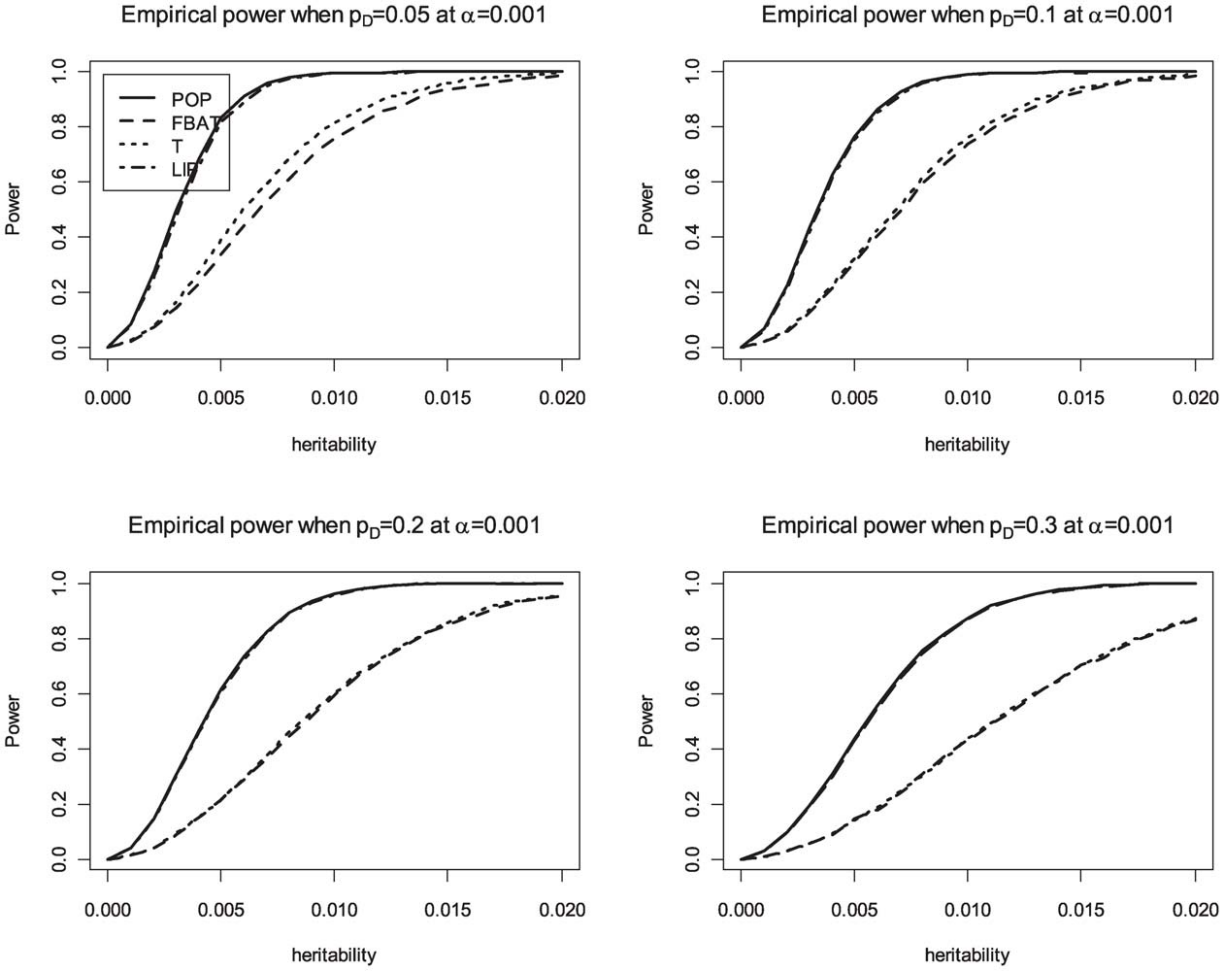
Empirical power at 0.001 significance level for dominant disease. POP is the empirical power of the standard population-based method. T is the empirical power of the Wald test based on the conditional mean model [22] for between-faimly components. LIP is the empirical power of the combined p-values with Liptak's method. In this figure, FBAT and T are completely overlapped.

**Figure 3:**
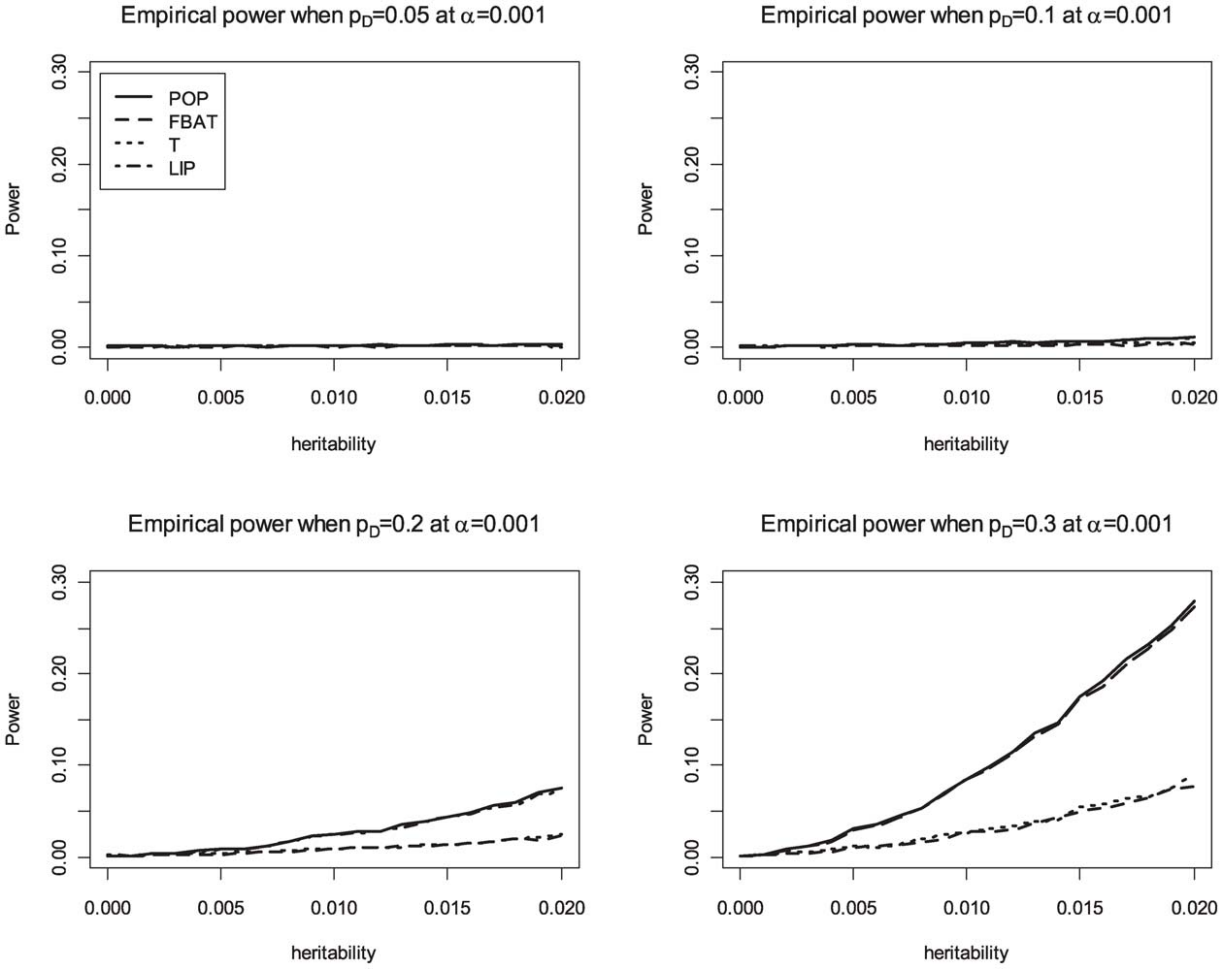
Empirical power at 0.001 significance level for recessive disease. POP is the empirical power of the standard population-based method. T is the empirical power of the Wald test based on the conditional mean model [22] for between-family components. LIP is the empirical power of the combined p-values with Liptak's method. In this figure, FBAT and T are completely overlapped.

For the analysis of quantitative traits, Table 4 provides the empirical power for 500K GWAS from 2000 replicates when there is no population stratification. Under the assumption of an additive disease model for a quantitative trait, the genetic effect, *a*, is given as a function of the heritability, *h* ^2^, the minor allele frequency *p_D_*_ı_ and the phenotypic variance, *σ* ^2^, by: *a* = *σh*/[2*p*(1−*p*)(1−*h* ^2^) ]^0.5^. In the simulation study, we assume heritabilities of *h* ^2^ = 0.001, 0.005, 0.01 and 0.015 for 2,000, 2,500 and 3,000 trios. The allele frequency of the disease locus, *p_D_*_ı_, is 0.3 and the phenotypic variance is 1. We compare the achieved power levels of the proposed overall family-based association test, *Z_i_*, with the weighting approach by Ionita-Laza et al [12], the original VanSteen approach [11], the QTDT approach [28] and population-based analysis, i.e. using linear regression of the phenotype *Y* on the genotype *X*. Bonferroni correction is used to adjust for multiple testing in the population-based analysis, FBAT, QTDT and the proposed method. The results in Table 4 suggest that the proposed association test achieves power levels that represent a major improvement over the existing methods for family-based association tests (VanSteen [11] or Ionita-Laza [12]). Our approach reaches the same power levels as the population-based analysis. For the power comparisons that are shown in Figure 1, Figure 2, and Figure 3, the number of trios is assumed to be 1,000 in 500K GWAS and the empirical powers are calculated based on 10,000 replicates at an *α*-level of 0.001 for the all genetic models. The results confirm that the Liptak's method combining *T_i_* and *FBAT_i_* has similar power to the population-based method, and the choice of equal weights performs well. The simulation results in Table 4 also suggest that QTDT [28] approach achieves similar power levels as the standard FBAT approach, which is consistent with previously reported findings in the literature [29]. However, both standard FBAT and QTDT are still much less powerful than the proposed overall family-based association test. Table 5 shows the empirical power for a GWAS with 100,000 SNPs in the presence of population stratification. For the parameters of this simulation study, we assume *F_ST_* = 0.001, 0.005, 0.01, and 0.05, and the additive mode of inheritance at the disease locus with values for the heritability of *h* ^2^ = 0.005, 0.01 and 0.015. The disease allele frequency *p_D_*_ı_ in the ancestral population is assumed to be 0.3. The phenotypic data is simulated so that their phenotypic means for two subpopulations are 0 and 0.2 respectively. Each individual/trio is assigned to either subpopulation with probability 0.5. The parental genotypes are used to estimate the ancestry for EIGENSTRAT as before. Various methods have been suggested to adjust the population stratification in a population-based designs and we compare the proposed methods with the EIGENSTRAT approach [10]. In order to maximize the power of the proposed method, we apply the EIGENSTRAT approach to the population-based component *T_i_* of our approach, i.e. principal component analysis based on the parental genotypes and the offspring's phenotype is integrated into the generalized Armitage test for *T_i_* [27]. To keep the power comparisons unbiased, the population-based components of the approaches by VanSteen and Ionita-Laza are also adjusted for population admixture, using the EIGENSTRAT approach. The results in Table 5 show that the proposed test statistic *Z_i_* is considerably more powerful than population-based analysis adjusted with EIGENSTRAT. QTDT is slightly more powerful than FBAT, but it is much less powerful than LIP as is in Table 4. This suggests that EIGENSTRAT should be applied only to between-family component in family-based association studies. Our unpublished work showed that the proposed approach can be less powerful than the combination of population-based analysis and EIGENSTRAT if *pT_i_* is calculated from the conditional mean model [11],[22] without adjusting population stratification.

**Table 4:** Empirical power for GWAS under no population stratification.

*N_trio_*	*h* ^2^	POP	FBAT	QTDT	LIP	VAN	ION
2,000	0.001	0.0000	0.0000	0.0000	0.0000	0.0000	0.0000
	0.005	0.0200	0.0025	0.0010	0.0185	0.0080	0.0130
	0.01	0.2085	0.0125	0.0180	0.1955	0.0990	0.1505
	0.015	0.5725	0.0765	0.0150	0.5350	0.3045	0.4515
2,500	0.001	0.0000	0.0000	0.0000	0.0000	0.0000	0.0000
	0.005	0.0385	0.0030	0.0030	0.0370	0.0155	0.0210
	0.01	0.3970	0.0430	0.0430	0.3760	0.2025	0.2960
	0.015	0.8135	0.1420	0.1790	0.7995	0.5525	0.7380
3,000	0.001	0.0000	0.0000	0.0000	0.0000	0.0000	0.0000
	0.005	0.0740	0.0020	0.0070	0.0675	0.0325	0.0495
	0.01	0.5720	0.0810	0.0855	0.5495	0.3175	0.4710
	0.015	0.9175	0.2665	0.3265	0.8980	0.7055	0.8630

Empirical powers are calculated from 2,000 replicates at the genome-wide significance level 0.05 from Bonferroni method under no population stratification. LIP stands for the proposed method using Liptak method to combine *pFBAT_i_* and *pT_i_*.VAN and ION indicate the VanSteen approach screening top 20 SNPs and Ionita approach using an exponential weighting scheme with partitioning parameters of *K* = 7 and *r* = 2 respectively. FBAT are the results only from the within-family component and POP is the standard population-based method.

**Table 5:** Empirical power for GWAS under population stratification.

*F_ST_*	*h* ^2^	FBAT	QTDT	LIP	VAN	ION	EIG
0.001	0.005	0.0000	0.0010	0.0083	0.0000	0.0000	0.0000
	0.010	0.0000	0.0030	0.1157	0.0826	0.1157	0.0579
	0.015	0.0000	0.0085	0.3884	0.2975	0.3471	0.2562
0.005	0.005	0.0000	0.0000	0.0083	0.0083	0.0083	0.0083
	0.010	0.0000	0.0020	0.0909	0.0579	0.0661	0.0661
	0.015	0.0083	0.0080	0.3223	0.2810	0.3140	0.1901
0.01	0.005	0.0000	0.0015	0.0000	0.0000	0.0000	0.0000
	0.010	0.0000	0.0010	0.0909	0.0826	0.0579	0.0331
	0.015	0.0083	0.0135	0.3636	0.2975	0.3388	0.2645
0.05	0.005	0.0000	0.0000	0.01653	0.0330	0.0248	0.0000
	0.010	0.0083	0.0035	0.0992	0.0744	0.0826	0.0165
	0.015	0.0165	0.0080	0.3140	0.2645	0.2727	0.2066

The number of trios, *N_trio_*, is assumed to be 1,000. Empirical powers are calculated from 2,000 replicates at the genome-wide significance level 0.05 from Bonferroni method under no population stratification. LIP stands for the proposed method using Liptak method to combine *pFBAT_i_* and *pT_i_*. VAN and ION indicate the VanSteen approach selecting top 20 SNP and Ionita approach using an exponential weighting scheme with partitioning parameters of *K* = 5 and *r* = 2 respectively. FBAT indicates the empirical power only from *FBAT_i_* and EIG indicates the empirical power from EIGENSTRAT.

### Applications to a genome-wide association in the Framingham Heart study

For the assessment of the severity of pulmonary diseases, the lung volume of air that a subject can blow out within one second after taking a deep breath is an important endo-phenotype. It is referred to as the forced expiratory volume in one second (FEV1). FEV1 is an important measure for lung function and we apply the proposed method to a family-based GWAS of FEV1. The proposed method is applied to 550K GWAS Framingham Heart Study (FHS) data set for FEV1, and then we confirm whether the selected SNPs are replicated in the British 1958 Birth Cohort (BBC), another population sample, as well as two samples of asthmatics in the the Childhood Asthma management program (CAMP) [30] and an Afro-Caribbean group of families from Barbados (ACG) [31]. In FHS, 9,274 subjects were genotyped and 10,816 subjects of those had at least one FEV1 measurement. Of the 8637 participants with genotyping and FEV1 measures, only those with a call rate of 97% or higher were included. We adjusted the covariates, age, sex and the quadratic term of height that are known to be associated with FEV1. For within-family components, the FBAT statistic for quantitative trait was applied. Markers were excluded from the analysis if the number of informative families was less than 20, or the minor allele frequency was less than 0.05. In total, 306,264 SNPs were used for analysis and, based on the number of SNPs, rank-based empirical p-values, *pT_i_*, and the genome-wide significance level was obtained with Bonferroni correction. When we let *n* and *n_inf_* be the total number of individuals and the number of informative trios respectively, *n_inf_*: (2*n*−*n_inf_*) are used for the weights of *Z_i_* because some of parental phenotypes are available.

Table 6 shows the p-values for the top 10 SNPs from the proposed method. In our analysis, the genome-wide significance level at 0.05 is 1.636×10^−7^ and our results show that only the first ranked SNP, rs805294, is significant at the genome-wide level 0.2 with Bonferroni correction. For rs805294, we also checked the significance in other data sets, BBC, CAMP [30] and ACG [31]. In CAMP, 1215 subjects in 422 families were genotyped and there are 488 informative trios for rs809254 and in ACG, there were only 33 informative trios (Table 7). In the BBC, 1372 unrelated subjects were genotyped with the Affymetrix chip and 1323 unrelated subjects genotyped with the Illumina chip. In CAMP and ACG, age, sex and the quadratic terms of heights were adjusted and in the BBC, age, sex, height, recent chest infection and nurse were adjusted. Table 7 also shows that rs805294 is significant and their directions are same for the considered studies except for the ACG sample. In particular, in the ACG study, the MAF of the SNP is different from other studies, which indicates a different local LD structure; The ACG sample is from an Afro-Caribbean population, contrary to the other studies which only include Caucasian study subjects. In addition, the ACG sample lacks statistical power for this particular SNP, i.e. there are only 33 informative trios in this sample. Thus, the inconsistent finding in the ACG study could be attributable to genetic heterogeneity, i.e. different local LD structure/flip-flop phenomena [32], or insufficient statistical power. For meta analysis, the sample sizes are used as weights for Liptak's method and we use 13∶13∶5∶1 = FHS∶BBC∶CAMP∶ACG as weights because the between-family information is used only for FHS. If the p-value from Illumina gene chip in BBC and the p-values from FHS, CAMP and ACG are combined, then the p-values by Liptak's method using proposed weights and Fisher's method are 1.534×10^−8^ and 1.081×10^−7^ respectively, and they become 4.625×10^−9^ and 3.554×10^−8^ if the p-values from one-tailed tests are used for BBC, CAMP and ACG with the same direction of FHS. If the p-value from the Affymetrix gene chip in BBC is combined with the other studies, then they are 3.787×10^−8^ (Liptak's method) and 1.890×10^−7^ (Fisher's method) for two-tailed tests, and 1.098×10^−8^ (Liptak's method) and 6.236×10^−8^ (Fisher's method) for one-tailed tests. As a result we can conclude that rs805294 is significantly associated with FEV1 at a genome-wide scale and the gene, LY6G6C, associated with rs805293 will be investigated in further studies.

**Table 6:** Applications to forced expiratory volume in one second in Framingham Heart study.

SNP	Chrom	Position	MAF	Num. Info. Fam.	*FBAT_i_*	*pT_i_*	*Z_i_*
rs805294	6	31796196	0.340	918	4.300×10^−3^	2.073×10^−5^	5.929×10^−7^
rs10863838	1	208750806	0.450	1016	7.408×10^−5^	2.535×10^−3^	2.553×10^−6^
rs6794842	3	119308208	0.331	950	3.226×10^−2^	2.400×10^−5^	6.654×10^−6^
rs804963	14	85918211	0.460	1031	9.786×10^−2^	2.775×10^−6^	7.060×10^−6^
rs525914	11	119200660	0.187	711	9.204×10^−4^	1.888×10^−3^	2.081×10^−5^
rs1886280	10	89347496	0.362	971	1.797×10^−2^	2.297×10^−4^	2.511×10^−5^
rs710469	3	188467212	0.491	1058	3.202×10^−3^	1.388×10^−3^	2.639×10^−5^
rs10799746	1	22497833	0.168	651	1.388×10^−2^	3.538×10^−4^	2.748×10^−5^
rs1225888	20	15972225	0.449	1007	7.518×10^−5^	1.736×10^−2^	2.994×10^−5^
rs4638547	15	71122046	0.377	999	3.454×10^−5^	2.760×10^−2^	3.549×10^−5^

The number of markers is 306,264 and the genome-wide significance level at 0.05 is 1.636 × 10^−7^. The top 10 SNPs from *Z_i_* are selected, assuming additive disease mode of inheritance. For *pT_i_*, the estimated powers are used and the weights for LIP are calculated with the number of informative trios.

**Table 7:** Descriptive statistics and results of rs805294 in different studies.

	FHS	British Cohort		CAMP	BAR
		Affy	Illumina		
Num. Info. Fam.	918	-	-	488	33
Sample Size	-	1372	1323	-	-
MAF	0.34	0.36	0.36	0.33	0.22
P-values	**−**5.929×10^−7^	**−**1.234×10^−2^	**−**6.534×10^−3^	**−**1.370×10^−2^	7.84×10^−1^

The negative sign of the P-values indicates that the minor alleles are under-expressed in cases.

## Discussion

Genome-wide association studies have become one of the most important tools for the identification of new disease loci in the human genome. However, even though advances in genotyping technology have enabled a new generation of genetic association studies that provide robust and replicable findings, population stratification/genetic heterogeneity and the multiple testing problems continue to be the major issues in the statistical analysis that have to be resolved in each study. While family-based association tests provide analysis results that are completely robust against confounding due to population-substructures, the analysis approach is not optimal in terms of statistical power. Numerous approaches have been suggested to minimize this disadvantage of family-based association tests but the previous approaches had to compromise either in terms of robustness or in terms of efficiency.

In this communication, we develop an approach that efficiently utilizes all available data, while maintaining complete robustness against confounding due to population substructure. The proposed methods combines the p-values of the family-based tests (the within-component) with the rank-based p-values for population-based analysis (the between component) to achieve optimal power levels. The use of rank-based p-values for the population-based component is similar in spirit to the genomic control approach. In principle, the genomic control functions as rescaling the variance inflated due to population stratification under the assumption of the constant *F_ST_*. Rank-based p-value directly rescales the statistics based on their ranks, which always generates the uniformly distributed p-value and provides validity even for varying *F_ST_* due to local population stratification etc.

Although our simulations are limited to independent unascertained samples and quantitative traits, the proposed work can be easily extended to ascertained samples, large pedigree, or different trait types, etc. By replacing the parental genotypes with the sufficient statistics by Rabinowitz&Laird [19], the FBAT-statistic and the screening-statistic can be adopted straight-forwardly to designs with extended pedigrees [23]. Similarly, parental phenotypes can be incorporated into the conditional mean model [23] or its non-parametric extensions [33] as additional outcome variables. The optimal weights can vary between the different scenarios and further theoretical investigation is currently ongoing, but limited initial simulation studies suggest that equal weights, while not always the most powerful choice in such situation, will always result in more powerful analysis than currently used methods.

## Supporting Information

Text S1 The validity of the proposed method.
(0.04 MB DOC)

## References

[1] Aulchenko YS, de Koning DJ, Haley C (2007). Genomewide rapid association using mixed model and regression: a fast and simple method for genomewide pedigree-based quantitative trait loci association analysis.. Genetics.

[2] Chen WM, Abecasis GR (2007). Family-based association tests for genomewide association scans.. Am J Hum Genet.

[3] Elston RC, Gray-McGuire C (2004). A review of the ‘Statistical Analysis for Genetic Epidemiology’ (S.A.G.E.) software package.. Hum Genomics.

[4] Lange C, Blacker D, Laird NM (2004). Family-based association tests for survival and times-to-onset analysis.. Stat Med.

[5] Laird NM, Horvath S, Xu X (2000). Implementing a unified approach to family-based tests of association.. Genet Epidemiol.

[6] Lange C, Silverman EK, Xu X, Weiss ST, Laird NM (2003). A multivariate family-based association test using generalized estimating equations: FBAT-GEE.. Biostatistics.

[7] Devlin B, Roeder K (1999). Genomic control for association studies.. Biometrics.

[8] Devlin B, Roeder K, Wasserman L (2001). Genomic control, a new approach to genetic-based association studies.. Theor Popul Biol.

[9] Pritchard JK, Stephens M, Donnelly P (2000). Inference of population structure using multilocus genotype data.. Genetics.

[10] Price AL, Patterson NJ, Plenge RM, Weinblatt ME, Shadick NA (2006). Principal components analysis corrects for stratification in genome-wide association studies.. Nat Genet.

[11] Van Steen K, McQueen MB, Herbert A, Raby B, Lyon H (2005). Genomic screening and replication using the same data set in family-based association testing.. Nat Genet.

[12] Ionita-Laza I, McQueen MB, Laird NM, Lange C (2007). Genomewide weighted hypothesis testing in family-based association studies, with an application to a 100K scan.. Am J Hum Genet.

[13] Murphy A, Weiss ST, Lange C (2008). Screening and replication using the same data set: testing strategies for family-based studies in which all probands are affected.. PLoS Genet.

[14] Nagelkerke NJ, Hoebee B, Teunis P, Kimman TG (2004). Combining the transmission disequilibrium test and case-control methodology using generalized logistic regression.. Eur J Hum Genet.

[15] Epstein MP, Veal CD, Trembath RC, Barker JN, Li C (2005). Genetic association analysis using data from triads and unrelated subjects.. Am J Hum Genet.

[16] Chen YH, Lin HW (2008). Simple association analysis combining data from trios/sibships and unrelated controls.. Genet Epidemiol.

[17] Zhu X, Li S, Cooper RS, Elston RC (2008). A unified association analysis approach for family and unrelated samples correcting for stratification.. Am J Hum Genet.

[18] Lange C, DeMeo DL, Laird NM (2002). Power and design considerations for a general class of family-based association tests: quantitative traits.. Am J Hum Genet.

[19] Rabinowitz D, Laird N (2000). A unified approach to adjusting association tests for population admixture with arbitrary pedigree structure and arbitrary missing marker information.. Hum Hered.

[20] Spielman RS, Ewens WJ (1998). A sibship test for linkage in the presence of association: the sib transmission/disequilibrium test.. Am J Hum Genet.

[21] Lange C, Laird NM (2002). On a general class of conditional tests for family-based association studies in genetics: the asymptotic distribution, the conditional power, and optimality considerations.. Genet Epidemiol.

[22] Lange C, Lyon H, DeMeo D, Raby B, Silverman EK (2003). A new powerful non-parametric two-stage approach for testing multiple phenotypes in family-based association studies.. Hum Hered.

[23] Lange C, DeMeo D, Silverman EK, Weiss ST, Laird NM (2003). Using the noninformative families in family-based association tests: a powerful new testing strategy.. Am J Hum Genet.

[24] Liptak T (1958). On the combination of independent tests.. Magyar Tud Akad Mat Kutato' IntKo''zl.

[25] Balding DJ, Nichols RA (1995). A method for quantifying differentiation between populations at multi-allelic loci and its implications for investigating identity and paternity.. Genetica.

[26] Cavalli-Sforza LL, Piazza A (1993). Human genomic diversity in Europe: a summary of recent research and prospects for the future.. Eur J Hum Genet.

[27] Armitage P (1955). Tests for linear trends in proportions and frequencies.. Biometrics.

[28] Abecasis GR, Cardon LR, Cookson WO (2000). A general test of association for quantitative traits in nuclear families.. Am J Hum Genet.

[29] Diao G, Lin DY (2006). Improving the power of association tests for quantitative traits in family studies.. Genet Epidemiol.

[30] (1999). The Childhood Asthma Management Program (CAMP): design, rationale, and methods. Childhood Asthma Management Program Research Group.. Control Clin Trials.

[31] Barnes KC, Neely JD, Duffy DL, Freidhoff LR, Breazeale DR (1996). Linkage of asthma and total serum IgE concentration to markers on chromosome 12q: evidence from Afro-Caribbean and Caucasian populations.. Genomics.

[32] Lin PI, Vance JM, Pericak-Vance MA, Martin ER (2007). No gene is an island: the flip-flop phenomenon.. Am J Hum Genet.

[33] Jiang H, Harrington D, Raby BA, Bertram L, Blacker D (2006). Family-based association test for time-to-onset data with time-dependent differences between the hazard functions.. Genet Epidemiol.

