# Supplementary material for: On the Analysis of Genome-Wide Association Studies in Family-Based Designs: A Universal, Robust Analysis Approach and an Application to Four Genome-Wide Association Studies
Source: PLoS Genet. 2009 Nov 26;5(11):e1000741. doi: 10.1371/journal.pgen.1000741 (PMC2777973; doi:10.1371/journal.pgen.1000741)
Supplement: Text S1 — The validity of the proposed method. (0.04 MB DOC) [file pgen.1000741.s001.doc]

**Text S1 The validity of the proposed method**

For simplicity we assume trio design with a single offspring even though it can be easily extended to general pedigree. If we let *P_ij_*_1_ (*P_ij_*_2_) be the parental genotypes/sufficient statistics at the *i*th marker in the *j*th trio, and we let *X_ij_* and *Y_j_* be the offspring genotype at the *i*th marker and phenotype for jth family respectively, the likelihood for the trio is

$l(X_{j},Y_{j},P_{ij1},P_{ij2})=l(Y_{j},P_{ij1},P_{ij2})+l(X_{j}|P_{ij1},P_{ij2},Y_{j})$.

In particular, because *FBAT_i_* is based on the *l*(*X_j_* | *P_ij_*_1_, *P_ij_*_2_, *Y_j_*) and *T_i_* is based on *l*(*Y_j_*, *P_ij_*_1_, *P_ij_*_2_), we can assume that *FBAT_i_* and *T_i_* are independent.

Now if we assume that *f*(*p*_1_, *p*_2_; *w*_1_, *w*_2_) is the method to combine p-values *p*_1_ and *p*_2_ with weights *w*_1_ and *w*_2_, the type I error of the combined p-value at significance level *α* can be expressed as follows:

$P\left[f(pFBAT_{i},pT_{i};w_{FBAT},w_{T})<\alpha\right]$.

For the Liptak’s method, *f*(*p*_1_, *p*_2_; *w*_1_, *w*_2_) is $\Phi(w_{1}Z_{p_{1}}+w_{2}Z_{p_{2}})$ and we assume that *pFBAT_i_* and *pT_i_* are the p-values from *FBAT_i_* and *T_i_*. Because the FBAT is robust to the population stratification, we can assume that *pFBAT_i_* follows uniform distribution under the null hypothesis. If we let *pT*_(1)_ < *pT*_(2)_ < … < *pT*(*_m_*) for GWAS with *m* markers, we can consider the rank-based p-values of each SNP for between-family component as follows:

$pT_{(i)}=\frac{i-0.5}{m}$.

Then the type I error in GWAS is

$$\begin{matrix} \frac{1}{m}E\left[\sum_{i=1}^{m} I\left\{\Phi(w_{FBAT}Z_{pFBAT_{i}}+w_{T}Z_{pT_{i}})<\alpha\right\}\right] \\ =\frac{1}{m}\sum_{i=1}^{m} P\left[\Phi(w_{FBAT}Z_{pFBAT_{(i)}}+w_{T}Z_{(i-0.5)/m})<\alpha\right] \end{matrix}$$

$$\begin{matrix} =\frac{1}{m}\sum_{i=1}^{m} P\left[w_{FBAT}Z_{pFBAT_{(i)}}+w_{T}Z_{(i-0.5)/m}<Z_{\alpha}\right] \\ =\frac{1}{m}\sum_{i=1}^{m} P\left[pFBAT_{(i)}<\Phi\left\{\frac{1}{w_{FBAT}}\left(Z_{\alpha}-w_{T}Z_{(i-0.5)/m}\right)\right\}\right] \\ =\frac{1}{m}\sum_{i=1}^{m} \Phi\left\{\frac{1}{w_{FBAT}}\left(Z_{\alpha}-w_{T}Z_{(i-0.5)/m}\right)\right\}. \end{matrix}$$

Because $pT_{(i)}=\frac{i-0.5}{m}$ and $1-pT_{(i)}=\frac{(m-i+1)-0.5}{m}=pT_{(m-i)}$, the following inequality guarantees that $\frac{1}{m}\sum_{i=1}^{m} \Phi\left\{\frac{1}{w_{FBAT}}\left(Z_{\alpha}-w_{T}Z_{(i-0.5)/m}\right)\right\}\leq\alpha$:

$f(\alpha)\equiv\Phi\left\{\frac{1}{w_{FBAT}}\left(Z_{\alpha}-w_{T}Z_{x}\right)\right\}+\Phi\left\{\frac{1}{w_{FBAT}}\left(Z_{\alpha}+w_{T}Z_{x}\right)\right\}-2\alpha\leq0$.

When *α* is less than or equal to 0.5, *f*(*α*) is maximized at *α* =0 or *α* =0.5 because $\frac{\partial^{2}}{\partial\alpha^{2}}f(\alpha)>0$. Also because *f*(0.5) = 0 and $\lim_{\alpha\to0} f(\alpha)=0$, $\frac{1}{m}E\left[\sum_{i=1}^{m} I\left\{\Phi(w_{FBAT}Z_{pFBAT_{i}}+w_{T}Z_{pT_{i}})<\alpha\right\}\right]$ ≤*α* is proven when *α* is less than 0.5 and larger than 0.
